# Rationale, Design, and Methods for *Nen Unkumbi/Edahiyedo (*“We Are Here Now”): A Multi-Level Randomized Controlled Trial to Improve Sexual and Reproductive Health Outcomes in a Northern Plains American Indian Reservation Community

**DOI:** 10.3389/fpubh.2022.823228

**Published:** 2022-07-13

**Authors:** Elizabeth Rink, Paula Firemoon, Michael Anastario, Olivia Johnson, Ramey GrowingThunder, Adriann Ricker, Malory Peterson, Julie Baldwin

**Affiliations:** ^1^Department of Health and Human Development, Montana State University, Bozeman, MT, United States; ^2^Fort Peck Community College, Poplar, MT, United States; ^3^AHC5, Robert Stempel College of Public Health & Social Work, Florida International University, Miami, FL, United States; ^4^Language and Culture Department, Fort Peck Assiniboine and Sioux Tribes, Poplar, MT, United States; ^5^School of Nursing, Johns Hopkins University, Baltimore, MD, United States; ^6^Center for Health Equity Research, Northern Arizona University, Flagstaff, AZ, United States

**Keywords:** community based participatory research, American Indian, sexually transmitted infections, teen pregnancy, sexual health education, culture, ecological systems theory, stepped wedge design

## Abstract

American Indian (AI) youth in the United States experience disproportionate sexual and reproductive health (SRH) disparities relative to their non-Indigenous, white counterparts, including increased rates of sexually transmitted infections (STIs), earlier sexual debut, increased rates of teen birth, and reduced access to SRH services. Past research shows that to improve SRH outcomes for AI youth in reservation communities, interventions must address complex factors and multiple levels of community that influence sexual risk behaviors. Here, we describe development of a multi-level, multi-component randomized controlled trial (RCT) to intervene upon SRH outcomes in a Northern Plains American Indian reservation community. Our intervention is rooted in a community based participatory research framework and is evaluated with a stepped wedge design that integrates 5 reservation high schools into a 5-year, cluster-randomized RCT. Ecological Systems Theory was used to design the intervention that includes (1) an individual level component of culturally specific SRH curriculum in school, (2) a parental component of education to improve parent-child communication about SRH and healthy relationships, (3) a community component of cultural mentorship, and (4) a systems-level component to improve delivery of SRH services from reservation healthcare agencies. In this article we present the rationale and details of our research design, instrumentation, data collection protocol, analytical methods, and community participation in the intervention. Our intervention builds upon existing community strengths and integrates traditional Indigenous knowledge and values with current public health knowledge to reduce SRH disparities.

## Introduction

Comprehensive national surveillance data on sexual and reproductive health (SRH) among American Indians (AI) is difficult to assess due to the underrepresentation of AIs in national surveys (1, 2). Extant studies demonstrate that AIs are disproportionately affected by adverse conditions attributable to SRH, compared to other populations (3, 4). AI youth report earlier onset of sexual intercourse than other adolescent populations in the U.S. (5, 6). The teen birth rate among AIs is 2 to 3 times higher than that among Caucasians (5, 7, 8). In addition, the rates of pre-term birth and low birth weight are much higher in AIs compared to Caucasians (9–12). STIs are up to 4.8 times higher for AI males and females compared to Caucasians, with young AI females having gonorrhea rates 6.6 times higher than white females (13). AI females have higher rates of miscarriages and ectopic pregnancies than Caucasian females and are at risk for infertility (14). For AI males, STIs can lead to urethritis, epididymitis, and prostate cancer, which is higher in AI men than all other racial and ethnic males in the U.S. (14). In addition, the costly and burdensome health consequences of AI youths' high STI rates include HIV and HCV infections. The estimated incidence rate of HIV in AI males and females is 1.97 times higher than in Caucasian males and females, and in recent years the incidence of HIV in AI males and females continues to rise (15). Furthermore, of all population subgroups in the U.S., the incidence of HCV is highest in AI groups (16).

In Montana, AI youth living on reservations experience disproportionate rates of sexually transmitted infections (STIs), early childbearing, and earlier sexual debut than their non-Indigenous peers at the state and national levels (17, 18). In 2019 in Montana, 54.6 percent of AI youth aged 15 to 18 reported ever having sexual intercourse in their life, compared to 42 percent of their white, non-Hispanic peers (17, 18). AI youth in Montana also experience higher rates of chlamydia (6,497 cases per 100,000 youth) as compared to white, non-Hispanic youth in the state (1,760 cases per 100,000 youth) (19). As of 2018, AI people accounted for 40 percent of reported gonorrhea cases and 23 percent of reported chlamydia cases in Montana, yet AI people make up only 6.6 percent of Montana's population (20, 21).

The literature related to SRH among AI youth identifies poverty, isolation, alcohol and other drug use, physical and sexual victimization, and lack of comprehensive and coordinated SRH education and clinical services as factors that influence unintended pregnancies and STIs (22–27). Evidence also suggests that ambivalence toward sex, social pressures, depression, anxiety, and experiences of historical trauma and loss influence sexual risk behavior among AIs (28–33). Mistrust of research and researchers continues in AI communities due to a legacy of neglect and betrayal by the U.S. government and by researchers from outside their communities (34–37). This mistrust impedes the cooperation between tribal communities and academics to design, implement, and evaluate effective interventions to address SRH disparities among AI youth (38, 39). Specifically in Montana, numerous socio-economic and environmental barriers contribute to SRH disparities for AI youth living on reservations across the state's sparsely populated Northern Plains, including lack of comprehensive SRH education in the schools, intergenerational historical trauma that impedes families and elders discussing topics related to SRH with young people, reduced access to comprehensive, evidence-based sexual and reproductive health (SRH) services, limited access to contraceptive options, and historic discrimination in healthcare settings (30, 40–43).

The complex factors that influence sexual risk behaviors among AI youth warrant novel AI community–specific interventions. Previous research with AI youth on SRH has lacked an ecological design and implementation, and it has neither addressed nor leveraged the interconnectedness of the individual, family, community, and larger systems in preventing STIs, HIV, HCV, and teen pregnancy (6, 44–46). For example, more research is needed into how the history of sexual trauma experienced by AI communities in the United States because of colonialization may be passed down through generations in families to impact family communication patterns about SRH, AI youth' sexual decisions, and their comfortable level accessing SRH services. Given the existing data that underscore the substantial SRH disparities among AI youth, there is an acute need for the rigorous testing of interventions using randomized controlled trial (RCT) designs in Indigenous communities (47–49).

Current intervention science research with Indigenous communities proposes further investigation in: (1) how to integrate diverse cultural belief systems, ecological perspective, and political contexts into RCTs; (2) how isolated communities with small populations that pose limits to statistical power, external validity, and generalizability assess scientific significance; (3) how effective implementation of RCTs can assess fidelity, acceptability, and sustainability; (4) how local ethical issues are conceptualized and addressed during RCT implementation with tribal members; and (5) how to effectively utilize Indigenous Research Methods (IRMs) and mixed methods in RCTs to decolonize research (50–53).

Furthermore, CBPR, which has become an established methodological framework for partnering with AI communities to conduct research, has been sparsely applied to understanding and addressing Indigenous SRH (54, 55). CBPR has the potential to facilitate the implementation and evaluation of an ecological intervention in AI communities, particularly given its amenability to multi-sectoral and multi-level stakeholder involvement in the research process (56–58). CBPR as a participatory framework for partnering with Indigenous communities has been identified as a methodological bridge to address the gaps in intervention science because it: (1) builds and maintains trust and reciprocity in community-academic partnerships; (2) empowers communities to address health disparities of importance to them in a culturally relevant manner; (3) unites the skills and knowledge of researchers with traditional and local knowledge and resources of the community to enhance research relevance to improve health; (4) increases community participation in research by involving community members; and (5) enriches data interpretation through the integration of community and academic expertise (59).

The aim of this paper is to describe the development of a CBPR multi-level, multi-component SRH intervention for AI youth. Our study, *NenUnkUmbi/EdaHiYedo* (“We Are Here Now,” or *NE*), is grounded in a 15-year collaborative research relationship between the Assiniboine and Sioux Tribes of the Fort Peck Reservation in Northeastern Montana and Montana State University (MSU) researchers to prevent STIs, HIV, HCV, and teen pregnancy among AI youth. *NE* is based on the Fort Peck Tribal Council's desire to implement a holistic, tribally driven SRH intervention for AI youth.

## Methods and Analysis

### Study Site

**Figure 1:**
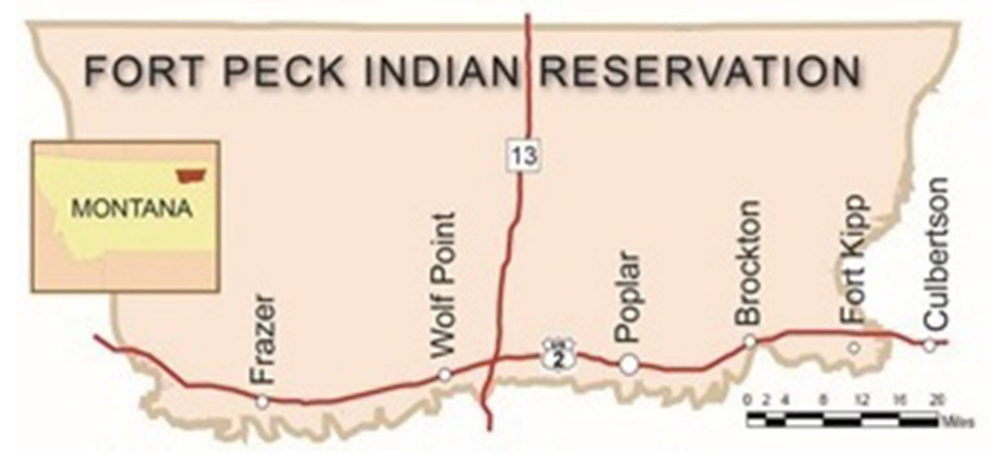
Study sites on or near the fort peck reservation.

*NE's* study site is the Fort Peck Indian Reservation (herein referred to as Fort Peck) in Northeastern Montana (Figure 1). Fort Peck is located in a Northern Plains frontier environment and spans 2.1 million acres. The reservation borders the 47$\frac{1}{2}$ parallel to the north (just south of the border with Canada), with Big Muddy Creek to the east, the Missouri River to the south, and Big Porcupine Creek to the west. Approximately 8,000 enrolled tribal members, predominately from the Assiniboine and Sioux Nations, live on the reservation. The Assiniboine and Sioux are descendants of the Nakoda, Nakota, Nakona, Lakota, and Dakota Nations. The Assiniboine comprise Wadopana (Canoe Paddlers Who Live on the Prairie) and Hudashana (Red Bottom) bands, and the Sioux comprise Sisseton/Wahpetons, the Yanktonais, and the Teton Hunkpapa bands (60).

*NE* is implemented in the communities of Frazer, Wolf Point, Poplar, Brockton, and Culbertson. These 5 communities have public high schools that serve Fort Peck tribal members. Frazer, Wolf Point, Poplar, and Brockton are on the reservation, and Culbertson is a border town on the east end of the reservation that AI youth from the reservation community of Fort Kipp attend.

Fort Peck tribal members between the ages of 14 and 18 experience poor SRH outcomes. Sixty-two percent of Fort Peck youth report being sexually active (61–63). For 2015-2017 Fort Peck Reservation had the second highest incidence rate of STIs in Montana (2159.2 cases per 100,000 people), a rate nearly 4 times higher than the rest of the state (64). The incidence of teen pregnancy among 14- to 18-year-old Fort Peck tribal members is 101.9 per 1,000 and is higher than the national average (61–63). Other problematic health trends among AI youth at Fort Peck include elevated rates of substance abuse, violence, depression, suicidal thinking, and self-reported suicide attempts that regularly exceed recent national base rates for other adolescent populations (65).

### Study Background and CBPR Foundation

*NE* utilizes a CBPR framework. *NE's* Principal Investigator (PI), Dr. Elizabeth Rink, began working with Fort Peck in 2006 when she was invited to Fort Peck by the then Fort Peck Tribal Health Director and council members from the Fort Peck Tribal Council to develop CBPR studies to address STIs among AI males. These early CBPR studies on AI males' SRH over a 5-year period led to the Fort Peck Tribal Council asking Rink to shift her focus from young adult AI males to developing an intervention for male and female AI adolescents to reduce SRH disparities among this population. The Council's primary request to Rink was that the intervention be holistic and involve youth, their families, and community members. This directive led to a four-year CBPR exploratory study and pilot intervention funded through the Center for American Indian and Rural Health Equity (CAIRHE) at MSU, collectively called the Fort Peck Sexual Health Study.

The Fort Peck Sexual Health Study used a CBPR framework and included a research team of tribal members working with Fort Peck Community College, outside researchers, and a community advisory board (CAB). The CAB was made up of five tribal representatives. CAB members included 3 females (2 Assiniboine and 1 Sioux) ages 30 to 50, and 2 males (1 Assiniboine and 1 Sioux) ages 45 to 55. The role of the CAB was to provide oversight and guidance for the exploratory study, the development of a pilot intervention, and ultimately the development and implementation of *NE*.

The Fort Peck Sexual Health Study was used to develop the research design for *NE*. The exploratory study used an explanatory sequential mixed methods design consisting of focus groups and interviews followed by a survey. Results from the exploratory study found: (1) family structures and values have shifted with sexual norms over time, changing how families communicate and address SRH; (2) youth culture surrounding sex is dissociated from the traditional cultural beliefs about sex, intimate relationships, and parenting held by elders; (3) social media impedes parents, elders, and youth from having thoughtful discussions about SRH; (4) SRH information received by AI youth was not associated with a decrease in sexual risk behaviors; (5) youth had positive attitudes toward pregnancy and positive familial support if they became pregnant; and (6) alcohol and substance use history, exposure to adverse life experiences, and increased levels of depression and anxiety were associated with increased likelihood of sexual risk behaviors (66). These results were then used to develop and implement a pilot intervention. Findings from the pilot intervention demonstrated increased condom use self-efficacy, increased condom use, and positive agreement with attitudes toward pregnancy among AI youth ages 14 to 18 years old. Our pilot intervention results also suggested: (1) parents needed their own education about SRH topics and strategies for speaking with their children about SRH; (2) increased interaction and communication with elders were desired by the youth; and (3) youth were not seeking SRH services for STI testing and birth control (66–68).

### Overview of *NenUnkUmbi/EdaHiYedo*

**Figure 2:**
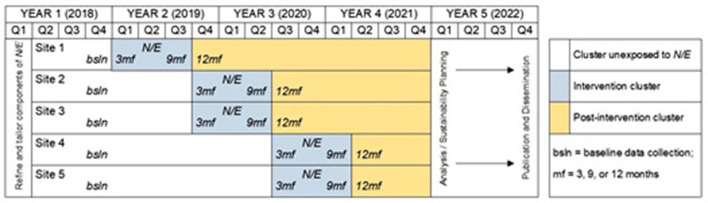
Trial design and timeline for implementing *NE* relative to grant and sites/schools.

Over a 6-month period, meetings among the CAB and the research team were held to review and discuss our qualitative and quantitative data on Fort Peck youth from our exploratory study and pilot intervention as well as existing literature on SRH interventions for youth. This series of meetings, in addition to the original direct request from the Fort Peck Tribal Council to Rink to develop and implement a holistic SRH intervention for youth on the reservation, resulted in *NE's* research design (Figure 2) (69).

To create a holistic intervention, *NE* was designed as a multi-level intervention. *NE's* different levels included: (1) A school-based SRH curriculum called *Native Stand*, designed to address individual-level factors that lead to sexual risk behaviors; (2) a family-level curriculum called *Native Voices*, tailored to increase communication between adult family members and youth about SRH topics; (3) a cultural mentoring component at the community level that pairs AI youth with adults and elders to discuss traditional AI beliefs and practices about SRH; and (4) a mobilizing strategy to activate a multi-sectoral network of youth-servicing organizations at the systems level in Fort Peck to coordinate SRH services for AI youth. *NE* uses a cluster-randomized stepped-wedge design (SWD), in which the 5 schools that AI youth from Fort Peck attend were the clusters randomized into the intervention 1 at a time, with all schools eventually being randomized to the intervention (70–74). The 5 schools are located in separate communities (Frazer, Wolf Point, Poplar, Brockton, Culbertson), mitigating the potential for cross-contamination between study sites. *NE* includes collaborations with Fort Peck Community College, the Fort Peck Language and Culture Department and the Fort Peck Tribal Epidemiology Team (Epi Team), which is a local committee that monitors infectious diseases on the reservation and the health status of tribal members; and the high schools of Frazer, Wolf Point, Poplar, Brockton, and Culbertson; and the Fort Peck Tribal Council.

**Figure 3:**
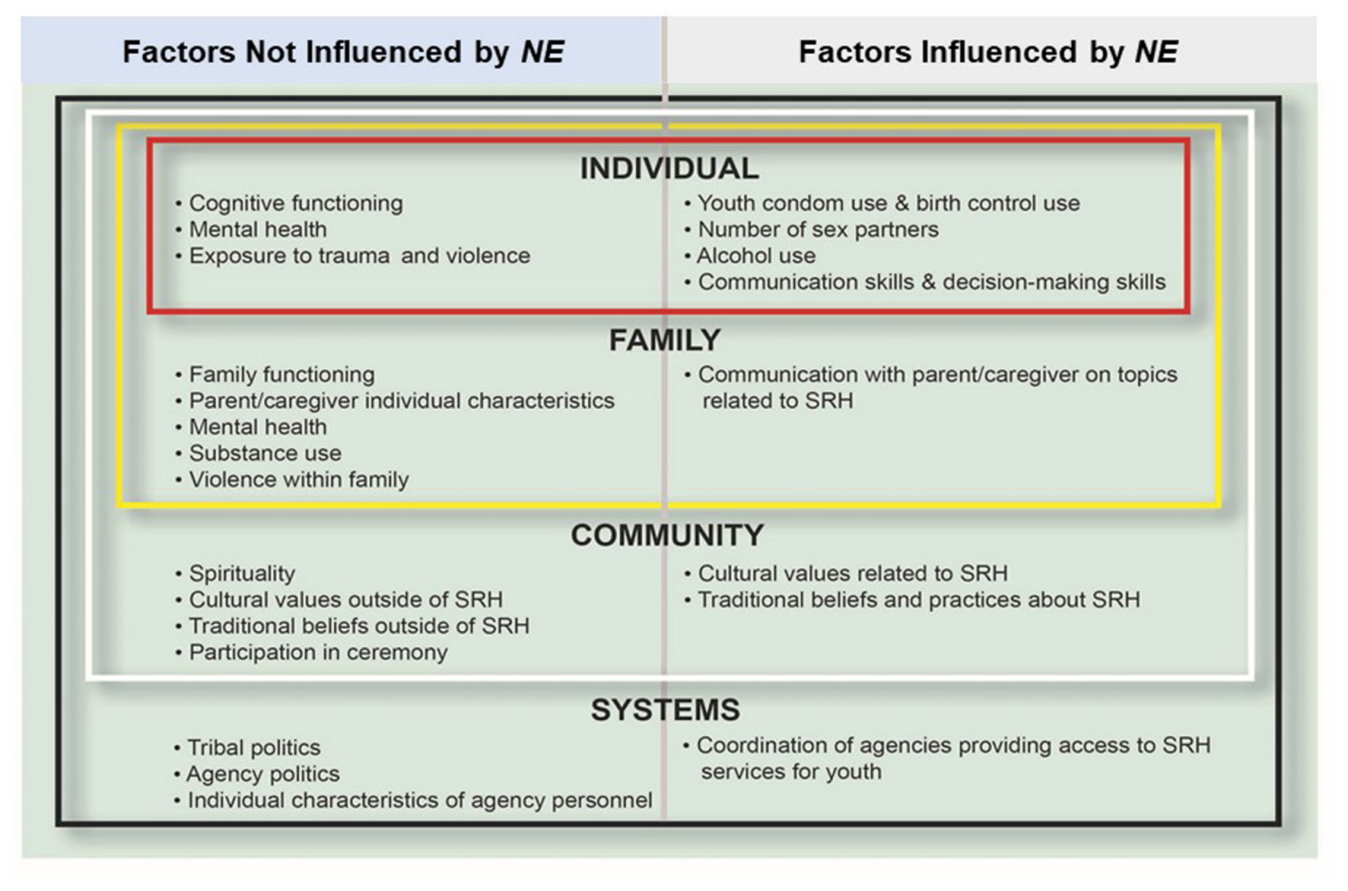
*NE*'s conceptual framework.

*NE* is designed using Ecological System's Theory (EST). EST had intuitive appeal for Fort Peck tribal members who have expressed an Indigenous worldview that emphasizes the interconnectedness of social, historical, cultural, spiritual, and kinship dynamics concerning SRH. Central to EST is an emphasis on the interaction of individual, family, social, cultural, and environmental factors to influence adolescents' behavior as they progress into young adulthood (75, 76). We specifically applied EST to assess our outcome variables, which included: (1) increased condom use, delayed onset of sexual intercourse, reduction in sex partners, increased contraceptive use, and reduction in substance use during sex (individual level); (2) increased youth–parent/legal guardian communication on SRH topics (family level); (3) increased cultural values and traditional beliefs about SRH topics (community level); and 4) increased coordination among education, health care, and social services on Fort Peck to provide SRH services for AI youth (systems level) (Figure 3).

#### Intervention Levels

*NE is* implemented simultaneously over the 9-month school year (Table 1). Components include:

**Table 1:** Overview of *NE*'s 9-month multi-level, multi-component intervention plan.

**Individual**	**Family**	**Community**	**Systems**
**Native Stand (NS):** 18-session school-based curriculum delivered twice a month over 9 months in *NE'*s 5 participating schools. NS will be integrated into the schools' existing class schedules.	**Native Voices (NV):** 4 sessions delivered once every other month to students and parents/legal guardians after school hours at *NE*'s 5 participating schools.	**Cultural Mentoring Program (CMP):** 9 mentoring sessions delivered twice a month over 9 months that include one group activity with CMP mentors and mentees and one 1:1 meeting. CMP activities will take place at varying locations on the Reservation depending on the activity.	**SRH Services:** Monthly meetings over 12 months with Epi Team at the Fort Peck Tribes Headquarters offices in Poplar, Montana
NS 1: Introduction to an overview of *NE* and Native Stand	NV 1: Introduction to *NE* and Native Voices	CMP 1: Assiniboine/Sioux kinship networks and concepts of family and genealogy	Epi Team meetings will take place monthly over 9 months to enhance coordination and implementation of SRH services for AI youth.
NS 2: Culture and tradition-part 1			
NS 3: Culture and tradition-part 2		CMP 2: Traditional male/female roles and cultural beliefs about healthy relationships	
NS 4: Healthy relationships-part 1			
NS 5: Healthy relationships-part 2	NV 2: Talking with youth about topics related to sex	CMP 3: Cultural beliefs about sex and how to make decisions about sex that integrate cultural values	
NS 6: Reproductive health-part 1			
NS 7: Reproductive health-part 2		CMP 4: Cultural beliefs about parenting and the role of family	
NS 8: Pregnancy and parenting			
NS 9: Preventing pregnancy	NV 3: Prevention of STIs, HIV/AIDS, and HCV	CMP 5: The role of ceremony in traditional Assiniboine/Sioux culture	
NS 10: Condoms and birth control			
NS 11: Sexually transmitted diseases		CMP 6: The role of sweat lodges in AI spirituality	
NS 12: HIV/AIDS and HCV			
NS 13: Drugs, alcohol, and sex	NV 4: Strategies for fostering healthy relationships in your child's intimate relationships	CMP 7: Honoring ceremonies in the lives of young men and women	
NS 14: Mental health and sex			
NS 15: Negotiation and refusal skills		CMP 8: Understanding the sacredness of the land	
NS 16: Decision-making			
NS 17: Effective communication		CMP 9: Buffalo harvest	
NS 18: Putting it all together			

##### 1) Individual Level

*Native Stand* is a school-based, 27-module STI-, HIV-, and teen pregnancy–prevention curriculum, originally developed for rural white youth and adapted in 2008 as a stand-alone SRH curriculum for AI youth. For *NE*, an adaptation of *Native Stand* was developed by the research team, CAB, and Fort Peck Language and Culture that reduced *Native Stand's* 27 modules to 18 modules and included specific community-relevant traditional and contemporary cultural lesson plans (47, 77). *Native Stand* is integrated into each of the 5 schools' participating in *NE* established curriculum and delivered by trained facilitators in a classroom setting as part of the students' regular class schedule.

##### 2) Family Level

*Native Voices* is a video-based HIV/STI-prevention intervention designed for AI youth to address condom use, negotiation skills, group discussion, and role playing. *Native Voices* was adapted from 1 module into 4 modules to involve parents/legal guardians, as recommended by the CDC (48). Our adaptation of *Native Voices* involved students with one of their parents/legal guardians to promote parent/legal guardian/youth discussions about preventing STIs, HIV, HCV, and teen pregnancy. *Native Voices* is delivered by a trained facilitator.

##### 3) Community Level

The third level of *NE* is a cultural mentoring component that pairs AI youth with older adults and elders to discuss traditional AI beliefs and practices about reproductive health. The mentoring program is based on Assiniboine and Sioux traditional knowledge and the National Mentoring Partnership standards for mentoring (78–80). We integrated the cultural mentoring program into *NE's* overall framework with an emphasis on addressing traditional topics related to SRH, such as overall information on cultural beliefs, values, and ceremonies, and more specific information on cultural beliefs and values regarding sex, having children, parenting, and relationships. Our cultural mentoring program includes older male and female tribal members paired with the youth by gender and tribal affiliation. AI youth meet one-on-one with their mentors each month and also monthly in mentor-mentee small groups. Depending on the purpose of the cultural mentoring sessions, the sessions are held at either the study's high schools, the Fort Peck Language and Culture Department's main office in Poplar, community centers on the reservation, or the Buffalo Ranch at Fort Peck.

##### 4) Systems Level

The fourth level of *NE* mobilizes the Epi Team to enhance the coordination and implementation of SRH services at Fort Peck. The members of the Epi Team utilize the recommendations in the CDC's *Contraceptive and Reproductive Health Services for Teens: Evidence-based Clinical Best Practices Guide* to enhance the coordination and implementation of SRH services for AI youth at Fort Peck (53, 59, 81). This enhancement process takes place during the Epi Team's monthly meetings by developing a coordination and monitoring plan that is tracked over time to address the barriers, facilitators, and solutions to increase access to SRH services for AI youth at Fort Peck.

#### Trial Design

A Stepped Wedge Design (SWD) trial is used to evaluate the effectiveness of NE. The SWD is a cluster-randomized trial, which involves random and sequential crossover of clusters from control to intervention until all clusters are exposed to the intervention (70–74). In *NE* the schools in Frazer, Wolf Point, Poplar, Brockton, Culbertson are the clusters randomized into the intervention. Our discussion to use SWD is consistent with tribal members' desires that all 14- to 18-year-old AI youth receive the intervention. In our SWD the first observation period corresponds to a baseline measurement observation, in which none of the clusters have been randomized to the intervention. Clusters are then randomized to the intervention at subsequent steps until all clusters have completed treatment.

Following randomization of the clusters to the intervention, *NE* is implemented and evaluated across three steps: Step 1 (1 school), Step 2 (2 schools), and Step 3 (2 schools). *NE* takes 9 months to implement in each cluster. To evaluate the intervention, the first observation period provides baseline measurements for all respondents drawn from every cluster. The cluster is then surveyed at 3, 9, and 12 months for the students in the cluster once they have begun participation in the intervention, and at baseline, 9 and 12 months for the parents/legal guardians in the cluster once they have begun participation in the intervention. Following completion of the intervention across all clusters, we will conduct the quantitative analysis. The SWD allows us to control for the effect of time and ensure that the full AI population of schools at Fort Peck receives *NE* (70–73). The systems-level component of *NE* is implemented reservation-wide rather than school-wide as the SWD is rolled out (82–85).

### Instrumentation

*NE's* data collection includes a student survey, a parent survey, and Epi Team tracking logs. *NE's* data collection instrumentation was designed in partnership with the study's CAB and collaborating tribal agencies using a combination of local and Indigenous knowledge about SRH topics and established measures.

To assess student participation in *NE*, we developed a student survey that draws from existing measures that have been implemented and validated in research studies with AI youth (22, 23, 47–50, 77, 86–92). Our primary outcome measure addresses condom use relative to the frequency of sexual intercourse. Secondary outcome measures address age of onset of sexual intercourse; the number of sex partners; frequency of sexual intercourse; pregnancy history; frequency and type of birth control used during sexual intercourse relative to the frequency of sexual intercourse; and frequency and types of substances used relative to the frequency of sexual intercourse. Tertiary outcome measures include parent/legal guardian communication about SRH topics, cultural identity, access to SRH services, and STI/HCV/pregnancy testing history. Other variables included in our student survey are attitudes, intentions, and skills related to SRH and mental health. Standard demographic measures in the student survey are age, gender, sexual orientation, school, and highest grade completed.

The parent/legal guardian survey is based on parents'/legal guardians' participation in *Native Voices*. It includes a list of SRH topics about which the parent/legal guardian have spoken to the child, as well as the frequency at which topics were discussed. The parent/legal guardian measures have been validated in previous studies evaluating parent/child SRH communication (91–93). Standard demographic measures included in the parent/legal guardian survey are age, gender, marital status, highest grade completed, and occupation.

The Epi Team's tracking logs monitor the progress toward completion of the 8 service domains (contraceptive access; provision of hormonal contraception/IUD; emergency contraception; cervical cancer screening; STI/HIV/HCV testing and treatment; cost, confidentiality, consent; health center infrastructure; and health center environment) for clinical practices outlined in the CDC's *Contraceptive and Reproductive Health Services for Teens: Evidence-based Clinical Best Practices Guide* (53, 59, 81). The Epi Team tracking log identifies the barriers, facilitators, solutions, and responsible personnel to increasing SRH service coordinate for tribal youth. The Epi Team tracking logs are measured qualitatively.

### Data Collection and Management

Data collection for the student and parent/legal guardian surveys are conducted on password protected iPads. The student and parent survey responses are then uploaded and housed on REDCap for data management (94, 95). Data collection for the students takes place during regular school hours. Data collection of the parents takes place individually either in their home or in an agreed upon location by the parent and the Fort Peck based research team. The data collection of the Epi Team tracking logs is conducted manually by the Epi Team member responsible for providing SRH services to AI youth on behalf of their agency. The tracking logs are collected quarterly by the MSU based research team during a regularly scheduled Epi Team meeting and uploaded to a secure server at MSU for organization.

### Recruitment

Students and their parent/legal guardian are recruited from each of the study's 5 schools. Based on discussions with school personnel and our previously successful recruitment strategies in our exploratory study and pilot intervention, recruitment occurs via flyers posted in the schools, letters to the parents, parent-teacher meetings, and word of mouth. Additionally, we hold a parent/legal guardian session at each of the study's 5 schools that provides an overview of *NE*, the content and format of the intervention components, and risks and benefits of participation. After written consent and assent is obtained from the parent/legal guardian and youth, respectively, they are enrolled in the study. Students and their parents/legal guardians, and students from each cluster, are recruited for participation at baseline. Students and parents/legal guardians are assigned unique study identification numbers. For the systems-level component of *NE*, only those staff members who sit on the Epi Team as representatives of their respective agencies participate in the study. Written informed consent is obtained from the head of each agency represented on the Epi Team and the staff member participating in the Epi Team.

### Incentives

Incentives for the students and parents/legal guardians are based on discussions with the CAB, research team members, and our prior experience implementing research studies at Fort Peck (96). Students receive $10 at the baseline, 3, and 9-month data collections and $20 at the 12-month data collection. Parents/legal guardians receive $10 at the baseline and 9-month data collections and at each of the 4 sessions of *Native Voices*, plus $20 at the 12-month data collection. A meal is also provided at the *Native Voices* sessions. Epi Team members do not receive incentives for participation in *NE*, as it is part of their tribal employment responsibilities. However, a meal is provided at the Epi Team meetings where the tracking logs are reviewed and discussed.

### Analysis Plan

NE's analysis plan uses a mixed methods approach. Student survey data will be evaluated using generalized linear mixed-effects models (GLMMs) for longitudinal data to model our outcome variables (97). The GLMM allows for individual-level binary and continuous responses to be modeled to control for baseline characteristics, autocorrelation within individuals, and clustering by school. We chose the GLMM approach for several reasons (98). First, analyses provide proper weighting when cluster sizes vary, which will be likely given the distribution of schools at Fort Peck (87–89, 97, 99). GLMMs are also the most frequently employed analytic method to adjust for the longitudinal nature of SWDs, and were chosen over the Generalized Estimating Equations (GEEs) because GEEs show inflated Type 1 error rates when the number of clusters is small, as is the case with the number of schools at Fort Peck (97). The GLMMs also allow us to model the unique effects of time that are evident in the SWD trial (this will be helpful, as sexual risk has been shown to vary over time in control group conditions at Fort Peck) (87–89, 97, 99).

Our quantitative data analyses also evaluate the influence of demographic factors, condom use intention, self-efficacy, refusal skills, knowledge, and motivation. Missing data is examined using multiple imputation, where missing values are replaced by a set of plausible values to generate a single estimate of the parameters of interest (100). Sensitivity analyses will be used to assess the robustness of primary results attributable to changes in assumptions regarding missing data by examining imputed and non-imputed data, adjusted for clustering (101, 102). STATA and R statistical software packages will be used to conduct the statistical analyses (103).

We estimate that a total of 456 students will be enrolled in *NE*. For our primary outcome variable, we expect to increase condom use as measured by the proportion of condom use relative to frequency of sexual intercourse from baseline to 12 months. Given our SWD, with an α of 0.05, power of 0.8, Nu = 292, DE_sw_ = 1.3273, and the expectation of 15% attrition during the study, a sample size of 456 will allow the detection of a 0.14 increase in the proportion of condom use relative to the frequency of sexual intercourse from baseline to the 12-month observation period (71–74).

For our systems level and the evaluation of the SRH services coordination on the reservation, we use qualitative data analysis. Epi Team tracking logs developed for *NE* based on the CDC's 8 service domains for improving SRH services for youth are transcribed and analyzed by coding for emergent themes that portray ongoing barriers and/or strengths in coordinating SRH services for Fort Peck youth. We sort the qualitative codes, relative to the CDC's 8 service domains. Then, within each service domain, we ascertain the axial and theoretical underpinnings relative to each. Atlas.ti software are used to develop open codes which are shared with the Epi Team and the CAB for discussion, refinement, and approval (104).

### Fidelity, Acceptability, and Sustainability

In addition to the actual intervention to measure SRH outcomes in AI youth*, NE* includes two qualitative methods of intervention fidelity and acceptability assessment to monitor and enhance the reliability and validity of the intervention. First, fidelity recommendations from the National Institutes of Health Behavior Change Consortium are implemented across 5 areas of study design and specifically include qualitative documentation of research team training, delivery of the intervention, receipt of the intervention, and enactment of intervention skills using Fidelity, Acceptability, and Sustainability (FAS) forms developed by the CAB and the research team members (82). Second, focus groups are an additional qualitative assessment to determine fidelity and acceptability, given the utility of focus groups in inductively determining key issues and ideas, their ability to elucidate process-oriented outcomes, and their use in previous SRH intervention evaluation studies (105, 106). We implement 4 to 8 focus groups at 12 months upon completion of the intervention in each cluster with students, parents/legal guardians, teachers and professionals from schools, agencies, and health care organizations.

The FAS forms and focus groups are analyzed using a grounded theory approach (107). All themes that emerge from the FAS forms and focus groups are reviewed, discussed, and agreed upon by the CAB. We will triangulate the qualitative data from our fidelity and acceptability strategies with the results from the data analyses of the primary, secondary, and tertiary outcomes to understand the value and effectiveness of *NE's* intervention levels (108–111). This analytical process will be conducted in iterative conversations between the study's CAB and research team members to reach consensus about *NE's* overall efficacy. Our overall goal is to use our findings to establish a culturally relevant sustainability plan for the integration of *NE* into extant Fort Peck infrastructures, and eventually replication *NE* in other tribal communities to prevent STIs, HIV, HCV, and teen pregnancy among AI youth (112–114).

## Ethics and Dissemination

### Ethical Approval

*NE's* ethical approval has had several steps. All of *NE's* research protocol s were reviewed and discussed amongst the research team members and the CAB for agreement and approval. *NE's* research protocols were then reviewed by the Fort Peck Institutional Review Board (IRB) for approval. Finally, the research protocols were sent to the MSU IRB as MSU policies require approval from a tribal IRB or tribal governance board, such as a tribal council, prior to the MSU IRB providing ethical approval for the study.

### Dissemination

The 15-year partnership between the Fort Peck Tribes and MSU researchers has resulted in numerous peer-reviewed publications, national presentations, and documentary films. Results of *NE* will continue our partnership's established record of disseminating research findings to scientific, professional, and community audiences. Work associated with *NE* will be presented at Fort Peck community meetings, to the Fort Peck Tribal Executive Board, on local radio, in the tribal newspaper, and presented to staff and faculty from the local high schools.

## Discussion

### Limitations

*NE* has anticipated limitations. First, our study relies on a number of widely-used self-reported measures of sexual risk behaviors, which may risk social desirability bias. We have successfully used these measures in our past exploratory studies at Fort Peck with AI youth and we are confident that they give accurate results of our outcome variables. Furthermore, our use of iPad increases perceived privacy. Previous studies of questionnaire administration modalities have documented that social desirability bias is a factor that may be mitigated by the use of password protected data collection devices when screening youth for health risk behaviors (115–117). Second, recruitment and retention of students may pose challenges, which we address by working closely with our CAB, all of whom have deep roots with the families and communities at Fort Peck. In addition, *NE's* Fort Peck based research team members are trusted tribal members with extensive experience working in the schools on the reservation and can work with school staff, youth, and families to recruit and retain participants. *NE's* Principal Investigator's 15 years of research experience with the Fort Peck Tribes is also an asset for addressing recruitment and retention. Despite recruitment and retention being a stated limitation, given the combined strengths of *NE's* Fort Peck- and MSU-based research team members and the CAB, we are confident in our ability to recruit and retain an adequate sample size to detect a treatment effect set at a power of 80%, thereby establishing the potential generalizability of *NE*. Third, potential contamination is possible to sites that have not yet entered or completed the intervention. However, we anticipate that cross-contamination will not occur because the 5 schools participating in *NE* are situated 15 to more than 30 miles from each other in distinctive, self-contained rural reservation communities. Fourth, under the SWD, there is the possibility that an unobserved 3-way interaction will exist for time by cluster by intervention. Based on our work with the Fort Peck community and the multiple factors we have documented as implicated in sexual risk-taking among youth on the reservation in our exploratory studies, we do not expect appreciable exogenous or endogenous temporal impacts on sexual risk outside of the intervention. The small likelihood of this possibility is outweighed by the Fort Peck Tribes' requirement that all youth from each of the communities be able to receive the full intervention. Hence our decision to use the SWD.

### Discussion

*NE* was designed as part of a long standing tribal-academic partnership between the Fort Peck Tribes and MSU at the bequest of the Fort Peck Tribal Council. *NE's* innovative design is the result of an integration of current academic knowledge with tribally specific data, cultural wisdom, and existing reservation resources.

*NE's* individual level utilizes an adaptation of Native Stand, an evidence based sexual health curriculum for AI youth. *Native Stand* has been adapted for use in other tribal communities as a stand-alone SRH curriculum for AI youth (47, 77). In *NE*, Native Stand is integrated into the study's multi-level design as part of a larger community-wide initiative to reduce SRH disparities among AI youth. Specifically, we reduced *Native Stand's* standard 28 modules to 18 with a focus on healthy relationships, decision making, mental health and substance use, and communication skills. *NE's* family level has adapted the *Native Voices* curriculum for parents/legal guardians which was originally designed by the Centers for Disease Control and Prevention (CDC) as a one-time STI/HIV prevention educational session for AI youth. In *NE* we have adapted *Native Voices* for parents/legal guardians based on recommendations by the CDC for parental adaptions to *Native Voices* that have not been previously undertaken with AI families in an RCT (48). *NE's* community level involves cultural mentors teaching youth participating in the intervention. Existing cultural mentoring programs for AI youth have primarily been designed to address substance use, juvenile delinquency, and suicide prevention (49, 50). *NE* fills a gap in the body of research on the use of older adults and elders to strengthen AI youths' understanding of their traditional beliefs and practices about topics related to SRH. *NE's* system level utilizes the CDC's *Contraceptive and Reproductive Health*. Services for Teens *Evidence-based Clinical Best Practices Guide* to evaluate the coordination of SRH services for AI youth in a tribal community (53, 59, 81). The CDC guide was developed based on research with non-Native SRH–serving agencies and provides recommendations for tribal communities that, to our knowledge, have not been used in an Indigenous community. The adaptions made to *Native Stand, Native Voices*, and the *CDC's Contraceptive and Reproductive Health Services for Teens: Evidence-based Clinical Best Practices Guide* in combination with the creation of a SRH cultural mentoring program were the result of collaborative efforts between the study's CAB and members of the Fort Peck and MSU research team members.

*NE's* methodological approach combines a cluster-randomized SWD within a CBPR framework. We use quantitative and qualitative methods to assess our outcome variables. Further, *NE* has a fidelity, acceptability, and sustainability component using a combination of evaluation forms to document how the intervention was implemented and focus groups to ascertain how participants experienced the intervention. *NE's* multi-level design is consistent with the emerging field of Indigenous intervention science. In a review of 21 RCTs, Dickerson et al. found that integration of cultural contexts and traditional knowledge with western scientific methodologies warranted qualitative and quantitative techniques as well as CBPR strategies to generate culturally adapted RCT designs with Indigenous communities in the United States (118). Additionally, Jerrigan et al. highlight the importance of developing evidence based multi-level intervention designs in partnership with tribal communities that address the historical and contemporary contextual issues in tribal communities shaping health disparities (119). Others suggest that multi-level interventions are needed to build local capacity that can effect systems and the interactions within systems in tribal communities to make structural and policies changes that can ultimately impact individual level health outcomes (120). By incorporating individual-, family-, community-, and system-level context-specific factors in AI youth's human ecology, *NE* is positioned to intervene in behaviors, situations, and structures that lead to SRH disparities in tribal communities. Our qualitative and quantitative techniques and the multiple ways in which we triangulate our findings also can contribute to new conceptual and analytical paradigms for analyzing the effectiveness of RCTs within the unique cultural context of tribal communities (121).

## Conclusion

*NE* seeks to understand the complex factors influencing SRH among AI youth to ultimately reduce SRH disparities in tribal communities. *NE* integrates current public health knowledge about how to prevent STIs, HIV, HCV, and teen pregnancy with traditional Assiniboine and Sioux cultural values and existing tribal resources and infrastructure. *NE* is consistent with Fort Peck tribal values, belief systems, and ways of life on the reservation; thus, it is more likely to result in decreased SRH disparities. The CBPR process by which *NE* was developed can be generalizable to other AI communities with similar values, belief systems, and ways of life. *NE* can also serve as an evidence-based, culturally adapted, and multi-level, multi-component SRH intervention for AI youth and families aiming to prevent and reduce STIs, HIV, HCV, and teen pregnancy in AI communities.

## Data Availability Statement

The original contributions presented in the study are included in the article/supplementary materials, further inquiries can be directed to the corresponding author.

## Ethics Statement

The studies involving human participants were reviewed and approved by Montana State University Institutional Review Board and Fort Peck Tribes Institutional Review Board. Written informed consent to participate in this study was provided by the participants' legal guardian/next of kin.

## Author Contributions

The authors of this manuscript were the original research team members that designed the randomized control trial presented in this manuscript. ER completed the writing of the manuscript. OJ, PF, AR, RG, MA, MP, and JB reviewed the manuscript and provided editorial and content specific feedback. MP conducted the formatting for the manuscript. All authors have reviewed the submitted manuscript and approve the manuscript for submission.

## Funding

The study presented in this manuscript was funded by NIH NIMHD, Award #R01MD012761. No-01. ER (Principal Investigator). The study's ClinicalTrials.gov number is: NCT03694418.

## Conflict of Interest

The authors declare that the research was conducted in the absence of any commercial or financial relationships that could be construed as a potential conflict of interest. The reviewer LT declared a shared affiliation with the author AR to the handling editor at the time of review.

## Publisher's Note

All claims expressed in this article are solely those of the authors and do not necessarily represent those of their affiliated organizations, or those of the publisher, the editors and the reviewers. Any product that may be evaluated in this article, or claim that may be made by its manufacturer, is not guaranteed or endorsed by the publisher.
